# MRC Centre Neuromuscular Biobank (Newcastle and London): Supporting and facilitating rare and neuromuscular disease research worldwide

**DOI:** 10.1016/j.nmd.2017.07.001

**Published:** 2017-11

**Authors:** Mojgan Reza, Daniel Cox, Lauren Phillips, Diana Johnson, Vaishnavi Manoharan, Michael Grieves, Becky Davis, Andreas Roos, Jennifer Morgan, Michael G. Hanna, Francesco Muntoni, Hanns Lochmüller

**Affiliations:** a John Walton Muscular Dystrophy Research Centre, MRC Centre for Neuromuscular Diseases, Institute of Genetic Medicine, Newcastle University, Newcastle upon Tyne, UK; b Dubowitz Neuromuscular Centre, University College London Great Ormond Street Institute of Child Health, London, UK; c National Hospital for Neurology & Neurosurgery, UCL, Queen Square, London, UK

**Keywords:** Neuromuscular Diseases, Biobanking, BBMRI, RD-Connect

## Abstract

Neuromuscular diseases are both genetic and acquired conditions resulting in progressive muscle weakness and wasting which lead to disability and reduced survival. The availability of high-quality human biomaterial is crucial to support biomedical research with potential applications at all stages of development, from molecular pathophysiology to drug discovery, clinical trials and evaluation of biomarkers. Although significant progress has been made over the last few years in the diagnosis of these rare conditions, the genetic defect and underlying pathological abnormality remain unknown in approximately 1/3 of cases. Moreover, to date no definitive cure is available for most neuromuscular disorders, nor are there sufficiently reliable and specific biomarkers to monitor disease progression and response to treatment. This is in part due to the rarity and genetic heterogeneity of neuromuscular diseases and the lack of access to patient samples. The availability of the national MRC Centre Biobank for Neuromuscular Diseases in Newcastle and London has addressed this bottleneck and supported neuromuscular research. Nine years after the establishment of the MRC Centre Biobank, many high profile research publications have highlighted the positive impact of neuromuscular biobanking for translational research and proven this facility to be a unique repository source for diagnostics, basic science research, industry, drug development, and therapy.

## Highlights

• MRC Biobank provides rare NMD samples and data available for researchers.
• Repository to enable diagnostics, basic science, drug development and therapy.
• Included in national and international networks through data linkage and ontologies
• Biobank supports natural history studies and clinical trials.

## 1 Introduction

**Fig. 1:**
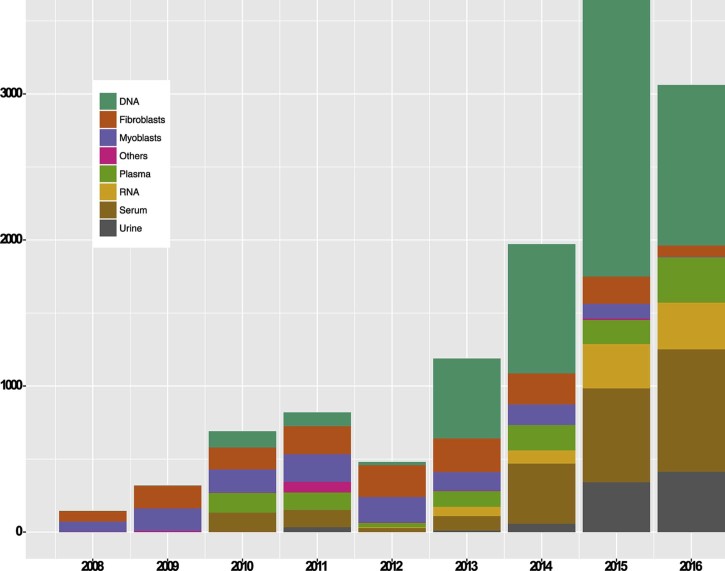
Sample types stored in the biobank. The Figure shows the type and number of stored samples available in both biobanks and their development over time. The number of collected biospecimens such as blood DNA and RNA, as well as, serum and plasma for biomarker studies were consistently increased due to routine sample collection in the neuromuscular clinics in both centres.

**Fig. 2:**
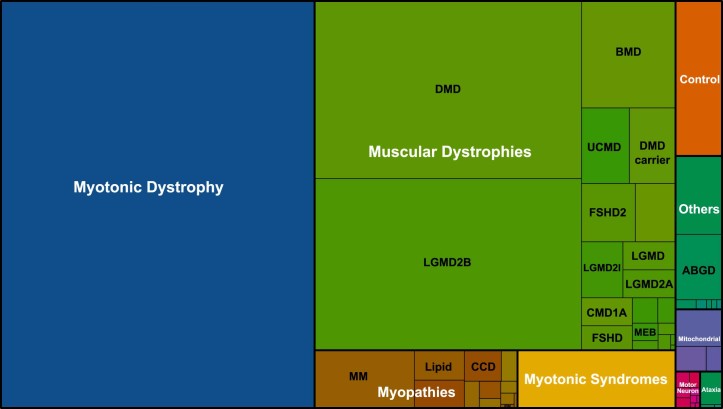
Classification of the diseases into large subgroups. Distribution of diseases among biobank samples. The size of the rectangles is proportional to the number of samples. Related diseases are summarised under generic terms in different colors. Undiagnosed samples are not shown.

NMDs represent a major cause of mortality and morbidity in children and adults. NMDs collectively affect an estimated 500,000 EU citizens and result in significant costs for families and healthcare systems. Many institutions and organisations, including the MRC Centre for Neuromuscular Diseases, have reduced the gap between science discoveries and patient benefit by establishing a multidisciplinary translational research activity in these disabling disorders. The MRC Centre for Neuromuscular Diseases is a partnership between the UCL Institutes of Child Health and Institute of Neurology, and the University of Newcastle upon Tyne and is one of the MRC's translational research centres in the UK. The MRC Centre for Neuromuscular Diseases Biobank is an integrated resource operating between Newcastle and London and builds one the five core activities of the MRC Centre for Neuromuscular Diseases to support the translational program of the centre. The biobank was established in 2008 and obtained funding by the MRC initially for five years (2008–2013) with an extension of a further five years until 2018. Both biobanks collect, preserve and distribute human samples to facilitate research in various areas of neuromuscular research. In 2010, the MRC Biobank was recognised as an official member of the EuroBioBank (http://www.eurobiobank.org/en/information/info_institut.htm) with a regularly published catalogue of samples made available for research. Currently, the MRC Biobank has assembled a collection of more than 13,000 anonymised rare disease samples mostly derived from NMD patients that are linked to patient data using a biobank ID and secure database. The collection includes DNA, muscle and skin biopsies, myoblasts, fibroblasts, urine, RNA, serum and plasma (Table 1, Fig. 1). Patient data are constantly updated against genetic reports. Samples are obtained from patients and family members with a confirmed or suspected diagnosis, such as muscular dystrophy, congenital myopathy, Charcot-Marie-Tooth disease and mitochondrial myopathy (Table 2 and Fig. 2). Notably, the collected samples can be requested by researchers carrying out studies on approval by the biobank's access committee. In addition, samples are distributed under a strict governance framework, with an internal application form and institutional Material Transfer Agreement (MTA) and only after having verified the ethical approval of the recipient laboratory for the planned study where required.

**Table 1:** Sample types stored in the biobank.

Sample type	London	Newcastle
CD133+ cells	12	0
Muscle derived fibroblasts	7	0
Fibroblasts	806	877
Myoblasts	681	713
Sorted CD56+ myoblasts	3	0
Frozen muscle	239	0
Frozen skin	7	0
Immortalised fibroblasts	1	27
Immortalised myoblasts	13	20
Peripheral blood lymphocytes	30	0
PBMCs	16	0
Plasma	245	789
Serum	303	2042
PBLs-transformed	55	0
Muscle derived pericytes	1	0
Synovial cells	10	0
Urine	74	777
DNA	126	4653
RNA	0	770
TOTAL	**2629**	**10668**

**Table 2:** Classification of the diseases.

Disease	Count	Disease	Count
Adult-Onset Cerebellar Ataxia Due To Cabc1/Adck3 Mutation	1	Limb-Girdle Muscular Dystrophy Type 2I	73
Adams Oliver Syndrome	2	Limb-Girdle Muscular Dystrophy Type 2K	18
Age-Related Macular Degeneration	10	Limb-Girdle Muscular Dystrophy Type 2L	26
Atypical Hemolytic-Uremic Syndrome	2	Lipid Storage Myopathy	60
Autosomal Dominant Motorneurone Disease	2	Marinesco-Sjögren Syndrome	6
Becker MD	403	MELAS Syndrome	4
Becker Muscular Dystrophy Female Carrier	32	MERRF Syndrome	7
Behr Syndrome	1	Minicore Myopathy With External Ophthalmoplegia	15
Benign Fasciculation Syndrome	1	Mitochondrial Complex I Deficiency	2
Brown-Vialetto-Van Laere Syndrome; Bvvls	4	Mitochondrial Complex II Deficiency	1
Bethlem Myopathy	126	Mitochondrial Complex III Deficiency	3
Central Core Disease	46	Mitochondrial Disorder	31
Carnitine Palmitoyltransferase II Deficiency, Infantile	1	Mitochondrial Myopathy	9
Centronuclear Myopathy	3	Morphea Sclerosis	1
Charcot-Marie-Tooth Ddisease (Not Specified)	1	Motor Predominant Axonal Peripheral Neuropathy	1
Charcot-Marie-Tooth Disease, X-Linked	2	Miyoshi Myopathy	231
Muscular Dystrophy, Congenital, 1C; MDC1C	20	Multi-Minicore Myopathy	2
Coenzyme Q10 Deficiency	2	Muscle Eye Brain Disease	18
Congenital Myopathy Due To SEPN1 Mutation	9	Multiple Acyl-Coa Dehydrogenase Deficiency	4
Congenital Myasthenic Syndromes	366	Muscular Dystrophy, Congenital Merosin-Deficient, 1A	57
Control	289	Myasthenia Gravis	2
Distal Myopathy	1	MYH7 Related Myopathy	6
DMD/BMD Intermediate	39	Myotubular Myopathy	20
DMD/BMD Intermediate Female Carrier	4	Myofibrillar Myopathy	12
Duchenne Muscular Dystrophy	1899	Myotonia Congenita	2
Diabetes-Deafness Syndrome, Maternally Transmitted	3	Myotonic Dystrophy Type 1	5116
Duchenne Muscular Dystrophy Female Carrier	140	Myotonic Dystrophy, Unspecified	4
Episodic Ataxia Type 2	2	Nemaline Myopathy	16
Emery-Dreifuss Muscular Dystrophy	19	Neuroferrinopathy	122
Enhanced S-Cone Syndrome	1	Neutral Lipid Storage Disease With Myopathy	2
Epilepsy	1	Non-Age Related Macular Degeneration	1
Facioscapulohumeral Muscular Dystrophy	50	Oculopharyngeal Muscular Dystrophy	6
Facioscapulohumeral Muscular Dystrophy Type 2	126	Oculopharyngodistal Myopathy	4
Friedreich Ataxia	28	Optic Atrophy	4
Hereditary Sensory And Autonomic Type 1	54	Optic Atrophy 1	20
Hereditary Inclusion Body Myopathy	1	Pompe Disease (Glycogen Storage Disease)	7
Idiopathic Pulmonary Arterial Hypertension	7	Progressive External Ophthalmoplegia (Polg2)	2
Inclusion Body Myositis	2	Retinitis Pigmentosa	12
Inherited Peripheral Neuropathy CMT2	3	Sjögren Syndrome	2
Inclusion Body Myopathy With Early-Onset Paget Kennedy Disease	32 1	Scoliosis	27
Leber Hereditary Optic Neuropathy	16	Ryanodine Receptor 1 Related Myopathy	8
Leukoencephalopathy With Vanishing White Matter	1	Severe Early-Onset Axonal Neuropathy Due To MFN2	3
Limb-Girdle Muscular Dystrophy (Not Specified)	59	Spinal Muscular Atrophy Type 1	9
Limb-Girdle Muscular Dystrophy Type 1B	8	Spinal Muscular Atrophy Type 2	6
Limb-Girdle Muscular Dystrophy Type 1C	8	Spinal Muscular Atrophy Type 3	7
Limb-Girdle Muscular Dystrophy Type 2A	59	Spinal Muscular Atrophy, Unspecified	6
Limb-Girdle Muscular Dystrophy Type 2B	1841	Spinal Muscular Atrophy With Respiratory Distress	1
Limb-Girdle Muscular Dystrophy Type 2C	10	Titinopathy/Myofibrillar Myopathy	8
Limb-Girdle Muscular Dystrophy Type 2E	2	Ullrich Congenital Muscular Dystrophy	19
Limb-Girdle Muscular Dystrophy Type 2F	1	Wolfram Syndrome 1	8

### 1.1 Ethical approval and governance arrangements

The ethics application for London has received a positive opinion by the ‘NHS National Research Ethics Service, Hammersmith and Queen Charlotte's and Chelsea Research Ethics Committee’ with REC reference number 06/Q0406/33, and for Newcastle by the ‘Newcastle and North Tyneside 1 Research Ethics Committee’ with REC reference number 08/HO906/28 + 5, which include consent and assent forms and patient information sheets adapted for different ages. These processes help to ensure that research is performed within the scope that is explained to the research participants and their carers. Transparent access arrangements are in place for use of the stored samples in research (see below).

### 1.2 Biobank samples, referral and consent

The samples are processed for storage with informed and signed consent of either the patients or their legal guardian, where an individual is considered unable to give consent. In addition, a referral form signed by the referring clinician accompanies each sample confirming the origin of the sample and includes core data including name, date of birth, gender, phenotype, ambulatory status, genetic results and confirmed or suspected diagnosis (Supplementary data S1–S4). Samples will not be processed and used in the absence of written informed consent. Patients participating in international studies, who do not speak English, need to be provided with information and consent forms in their own language which are provided in the research protocols of such studies. Muscle biopsies acquired for biobanking purposes are ‘left overs’ to diagnostic requirements and are collected at the time of routine procedures indicated by clinical need. Skin biopsies, blood, urine or other relevant fluids are mostly attained during routine clinical consultations in our centres, or if possible, opportunistically during the muscle biopsy procedure. Moreover, samples may be collected at clinical appointments as part of clinical trials and studies. Body fluid collection for biomarker studies including whole blood and urine are an integral component of the biobank, in order to address the lack of access to patient's biomaterial for both academic research and industry. Specimens from patients in various stages of disease, as well as, samples derived from control donors are collected in the search for biomarkers, as predictors, biological indicators or tests that will monitor disease progression. Similarly, routine whole blood collection and subsequent DNA/RNA extraction facilitates gene discovery in patients with neuromuscular conditions and unknown genetic defects. In addition to locally collected samples, various biomaterials may be obtained from other centres, both in the UK and abroad.

### 1.3 Submission of samples to the biobank from other centres

The majority of sample submission comes from local services and from studies carried out in London and Newcastle. In addition**,** the MRC Biobank encourages submission of biological samples from elsewhere, in particular samples obtained through multi-centre studies including natural history studies, clinical trials and collaborative studies with industry. The same strict, ethical and legal principles as for the local submissions are applied to the external submissions. External samples and patient data are held in strict confidence and identifiable information is not made available to anyone outside of the biobank. Standard Operating Procedures (SOPs) on sample collection, handling, sampling conditions for each type of sample and advice on material transport are provided by the biobank staff. The biobank supports researchers in developing study-specific protocols and SOPs and offers scientific advice for the best choice and use of biomaterials for their studies. Generally, shipment of samples and shipping cost is the responsibility of the sender, unless otherwise agreed with the biobank.

### 1.4 Disease classification

Biobanks both in Newcastle and London, collectively preserve samples deriving from more than 100 different conditions with known genetic defects. As shown in Fig. 2, a superordinate classification of the diseases includes myotonic dystrophy with more than 5000 samples, followed by muscular dystrophy samples and other rare diseases such as mitochondrial disorders, ataxia, motor neuron disease, congenital myopathy and myasthenic syndrome. A precise classification of subgroups is outlined in Table 2.

### 1.5 Sample handling and data management

Samples transported to Newcastle or London are processed using Standard Operating Procedures (SOPs) adapted from current EuroBioBank SOPs or protocols developed in the biobanks. The required data are recorded in the respective individual databases and stored samples are identified by a ‘linked-anonymised’ unique laboratory code which is allocated to each sample upon arrival, linking samples and clinical information to patients. In order to protect patient's privacy, all identifying information is held in a locked and access-restricted filing cabinet. Only authorised biobank staff has access to the ‘key’ for relinking the participants' identifying information with their data and samples. It is necessary to retain this link with identifying information to allow follow-up of research findings or feedback relevant, new information such as a confirmed diagnosis to the patients via their referring clinicians.

### 1.6 Catalogue of samples

Collected samples with a confirmed diagnosis are available to users via EuroBioBank catalogue (http://www.eurobiobank.org/en/services/Catalogue Home.html). The catalogue lists available samples by type of biomaterial or disease name and code according to ICD-10 [1], OMIM [2] and Orphanet [3] Classification. Once a sample has been located in the catalogue, it can be requested from the biobank in Newcastle or London. Additional clinical and genetic information including mutation, age and other relevant data -as far as available- can be requested from the biobank team. The MRC Biobank is fully incorporated in international biobanking frameworks such as RD-Connect [4], BBMRI-ERIC [5], [6] and EuroBioBank [7]. RD-Connect is a unique global infrastructure project that connects biobanks, registries, databases and clinical bioinformatics data used in rare disease research into a central resource for researchers worldwide. BBMRI-ERIC (Biobanking and Biomolecular Resources Research Infrastructure – European Research Infrastructure Consortium) is an infrastructure with sustainable funding from European member states covering a wide spectrum of biobanks including bioresources for all diseases, irrespective of whether they are considered common or rare, as well as population-based cohort studies. Within RD-Connect and in partnership with BBMRI-ERIC, a new online RD-Connect sample catalogue for rare disease biobanks has been developed. The catalogue is based upon the MIABIS standard and provides users with a searchable and filterable sample dataset, assembled from multiple rare disease biobank sample collections, making biobank data FAIR (Findable, Accessible, Interoperable and Reusable) [8]. The new online catalogue provides researchers with a more detailed and accurate collection of biobank sample data, giving users the ability to filter multiple sample data fields or to search for available biomaterials using specific terms such as disease name or classification codes.

### 1.7 Eligibility, access and application process

Samples are accessible to research groups based in the UK and elsewhere around the world. Applicants should be employees of a recognised academic, research or clinical institution; or employed or contracted by a commercial organisation working towards developing diagnosis and treatment for neuromuscular or other rare disorders. Samples can also be made available to scientists who are interested in basic science research i.e. in research of muscle structure and function. Researchers who wish to access the collection should initially contact the biobank coordinator directly. An ‘Application for use of samples for research’ form (Supplementary data S6–S8) provided by the biobank in Newcastle or London, should be completed by applicants giving a brief outline of the proposed study and for what purpose the samples will be used, followed by the number and type of samples required. Two members of the access committee will assess the suitability of the application and the biobank coordinator or relevant biobank member of staff will respond to the applicant. Upon approval of the application form, the applicant will be required to complete a MTA form and agree to the conditions of access set out within the agreement and return a signed e-copy to the Intellectual Property and Legal Services Department at Newcastle University or UCL. Samples supplied to the applicant from the biobank collection can only be for the purposes stated in the application form, accepted by the access committee and described in the MTA. Research publications or presentation using data or samples from the biobank should include an acknowledgement to the MRC Centre Biobank in Newcastle or London. These publications must also be reported to the biobank coordinator for reporting purposes. The applicant may be asked to cover the costs of retrieving, processing and dispatching samples.

### 1.8 Organisation, responsibilities and administration of the biobank

Upon receipt of the full application, a member of biobank staff will check to ensure that the samples requested are available and that all required information has been supplied by the applicant. If samples are not available the applicant will be notified and possible alternatives discussed. If any information is missing from the application the applicant will be asked to supply this before the application will be considered further. Access to samples is overseen by an access committee which is composed of the investigators of the MRC Centre for Neuromuscular Diseases (http://www.cnmd.ac.uk/research/research_groups). The function of the access committee is to ensure that measures are in place to achieve compliance with the agreed procedures, protect the interests of patients and the biobank, and to oversee sample provision for research purposes. It does not act in as a scientific review board. The access committee reviews the eligibility of the proposed study and decide whether information provided by the applicants would justify releasing samples, some of which are rare or even unique. The committee also oversees whether the applicant of a research project is an approved and recognised researcher. The laboratory team is responsible for implementation and day to day running of the biobank including processing and archiving samples, recording data, establishing primary cultures, extracting DNA and preparing samples for shipment. The team also acts as a central contact for distribution of material and data as required by project partners and responsible for maintaining stocks of biological material, records and datasets, as well as undertaking changes to database, protocols and SOPs. Additionally, the laboratory team undertakes regular quality control and maintenance of laboratory equipment to safeguard the quality of samples. Ethical issues are processed by the biobank coordinator who assists with relevant ethical approval and procedures. The coordinator facilitates and coordinates the daily clinical trial activities in collaboration with legal departments and sponsors.

### 1.9 National and international networking and sample distribution

**Fig. 3:**
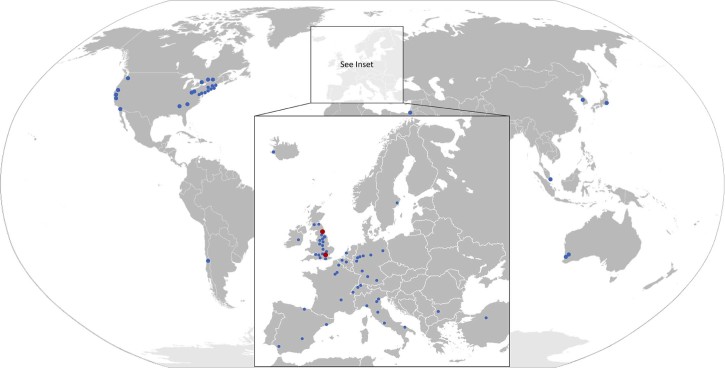
Distributed samples within the UK and worldwide. Worldwide distribution of Newcastle MRC Centre Biobank samples. Countries that have received samples from the biobank include the UK, USA, Japan, Australia, Spain, France, Germany and Italy.

Many research programs have benefited from specimens provided by the MRC Biobank. More than 700 research projects outside the MRC Centre have received samples for research confirming the national and international science resource and collaborative links of the MRC Centre. Fig. 3 shows research centres that have received samples from both, Newcastle and London biobanks. The MRC Biobank has also been critical in supporting research activities through the collection of samples for externally funded projects such as Optimistic and NeurOmics, natural history studies and clinical trials. Natural history studies and clinical trials supported by the biobank are listed in Table 3. On request and depending on the individual study or collaboration, the biobank principal investigators may assist with advice on grant writing procedures for external projects that involve the biobank.

**Table 3:** Examples of clinical trials and natural history studies supported by the biobank.

Study	Time period	Description
**Vision DMD**	2017–present	A phase IIb randomized, double-blind, placebo- and active-controlled study to assess the efficacy and safety of VBP15 in ambulatory boys with Duchenne muscular dystrophy
**AMO Pharma (NCT02858908)**	2016–present	A single-blind, phase 2 study to evaluate the safety and efficacy of tideglusib 400 mg Or 1000 mg for the treatment of adolescent and adult congenital and juvenile-onset myotonic dystrophy
**GNE Phase 3b (NCT02736188)**	2016–present	A phase 3b open-label extension study to evaluate the safety and efficacy of aceneuramic acid extended-release (Ace-ER) tablets in patients with GNE or hereditary inclusion body myopathy
**Disease Translation in DMD**	2016–present	Neuromuscular Rare Disease Translational Research in patients with Duchenne muscular dystrophy
**Pfizer DMD Study**	2015–present	Phase 1b/2, double-blind, dose escalation study to evaluate the safety and efficacy of PF-06252616 administered to ambulatory boys with Duchenne muscular dystrophy
**BMD Natural History Study (NCT01539772)**	2015–present	Becker muscular dystrophy – a natural history study to predict efficacy of exon skipping
**Testosterone Study (NCT02571205)**	2015–present	Observational outcomes in testosterone treatment of pubertal delay in Duchenne muscular dystrophy
**GNE SAER (NCT02377921)**	2015–present	A phase 3 randomized, double-blind study to evaluate the efficacy and safety of sialic acid extended release tablets in patients with GNE or hereditary inclusion body myopathy
**PhenoDM1 (NCT02831504)**	2015–present	PhenoDM1 - myotonic dystrophy type 1 (DM1) deep phenotyping to improve delivery of personalised medicine and assist in the planning, design and recruitment of clinical trials
**GNE Natural History Study (NCT01784679)**	2014–present	Hereditary inclusion body myopathy-patient monitoring program (HIBM-PMP): a registry and prospective observational natural history study to assess HIBM disease
**NeoGAA Extension**	2014–present	An open-label, multicenter, multinational extension study of the long-term safety and pharmacokinetics of repeated weekly infusions of NeoGAA in patients with pompe disease
**FSHD NH Study**	2014–present	A multi-centre collaborative study on the clinical features, expression profiling, and quality of life of infantile onset facioscapulohumeral muscular dystrophy
**OPTIMISTIC**	2014–present	Observational prolonged trial in myotonic dystrophy type 1 to improve quality of life standards
**SKIP-NMD**	2014–2016	A 2-part, randomized, double-blind, dose-titration, safety and tolerability study (part 1) followed by an open-label efficacy and safety evaluation (part 2) of SRP-4053 in patients with Duchenne muscular dystrophy amenable to exon 53 skipping
**FOR-DMD (NCT01603407)**	2013-present	An international, multi-centre study to compare the benefits and side effects of the three most widely prescribed steroid treatments in children with Duchenne muscular dystrophy
**Natural History of Dysferlinopathy (NCT01676077)**	2012-present	A collaborative project co-ordinated via the TREAT-NMD network and the Jain foundation and utilising the strengths and well defined populations already characterised in Europe and the US
**DMD Heart Protection Trial**	2010–2018	A double-blind, randomised multi-centre, placebo-controlled trial of combined ACE-inhibitor and beta-blocker therapy in preventing the development of cardiomyopathy in males with DMD without echo-detectable left ventricular dysfunction
**CMT Natural History Study**	2009–2019	Charcot-Marie-Tooth disease and related disorders: a natural history study
**AFM Natural History study** **(NCT 02780492)**	2009–2016	Developing tools for assessing the natural history of ambulant and non-ambulant DMD individuals to assist in antisense oligomer clinical trials
**PTC124 Trial**	2009	A phase 2a study of ataluren (PTC124) in non-ambulatory patients with nonsense–mutation-mediated Duchenne/Becker muscular dystrophy
**AVI-4658 Trial (phase 1)**	2009	Safety and efficacy study of antisense oligonucleotides in Duchenne muscular dystrophy

### 1.10 Gene discovery

Novel gene identification is supported by the MRC Biobank through the collection of blood samples and by supplying high quality DNA for whole exome (WES), whole genome (WGS) and Sanger sequencing. By the same token, new variants responsible for NMDs have been identified and published through use of samples derived from patients. For instance, mutations in *DPAGT1*, *ALG14* and *ALG2* have been shown to be causative for different subtypes of congenital myasthenic syndrome and the importance of asparagine-linked protein glycosylation for proper functioning of the neuromuscular junction [9]. Detection of mutations in *ANO10* indicates that *ANO10* defects cause secondary low *COQ10* and it has been proposed that *SCAR10* patients may benefit from *COQ10* supplementation [10]. Mutations in the Synaptotagmin 2 C2B domain represent an important cause of presynaptic congenital myasthenic syndromes and link them with hereditary motor axonopathies [11]. Two missense mutations residing in the N-terminal agrin domain were identified which resulted in reduced acetylcholine receptors clustering activity of agrin in vitro [12]. A publication has shown that homozygous missense mutations in *EXOSC8* cause progressive and lethal neurological disease in 22 infants from three independent pedigrees [13]. Recessive and dominant mutations in *COL12A1* have shown to cause a novel EDS/myopathy overlap syndrome in humans and mice [14]. It has been reported that mutations in the *MYO9A* gene may affect the presynaptic motor axon in patients with congenital myasthenic syndrome [15]. Table 4a and 4b highlights some more examples of genes and their functionality, which have been identified using biobank samples.

**Table 4:** Publications on gene discovery and function using biobank samples.

Title (gene discovery)	Reference	Journal
Identification of mutations in the MYO9A gene in patients with congenital myasthenic syndrome	(O'Connor, Topf et al. 2016)	Brain
Loss-of-function mutations in SCN4A cause severe foetal hypokinesia or ‘classical’ congenital myopathy	(Zaharieva, Thor et al. 2016)	Brain
Dihydropyridine receptor (DHPR, CACNA1S) congenital myopathy	(Schartner, Romero et al. 2016)	Acta Neuropathol.
Novel mutations expand the clinical spectrum of DYNC1H1-associated spinal muscular atrophy	(Scoto, Rossor et al. 2015)	Neurology
Phenotypic and molecular insights into spinal muscular atrophy due to mutations in BICD2	(Rossor, Oates et al. 2015)	Brain
A large deletion affecting TPM3, causing severe nemaline myopathy	(Kiiski, Lehtokari et al. 2015)	JND
ANO10 mutations cause ataxia and coenzyme Q10 deficiency	(Balreira, Boczonadi et al. 2014)	J Neurol.
Synaptotagmin 2 mutations cause an autosomal-dominant form of lambert-eaton myasthenic syndrome and nonprogressive motor neuropathy	(Herrmann, Horvath et al. 2014)	Am J Hum Genet.
Agrin mutations lead to a congenital myasthenic syndrome with distal muscle weakness and atrophy	(Nicole, Chaouch et al. 2014)	Brain
EXOSC8 mutations alter mRNA metabolism and cause hypomyelination with spinal muscular atrophy and cerebellar hypoplasia	(Boczonadi, Muller et al. 2014)	Nat Commun.
Mutations in the mitochondrial citrate carrier SLC25A1 are associated with impaired neuromuscular transmission	(Chaouch, Porcelli et al. 2014)	JND
Loss of function mutations in MICU1 cause a novel disorder affecting brain and muscle and reveal primary alterations in mitochondrial Ca2+ signalling as a new disease mechanism	(Logan, Szabadkai et al. 2014)	Nat Genet.
Treatable childhood neuronopathy caused by mutations in riboflavin transporter RFVT2	(Foley, Menezes et al. 2014)	Brain
COX10 mutations resulting in complex multisystem mitochondrial disease that remains stable into adulthood	(Pitceathly, Taanman et al. 2013)	JAMA Neurol.
Identification of KLHL41 mutations implicates BTB-kelch-mediated ubiquitination as an alternate pathway to myofibrillar disruption in nemaline myopathy	(Gupta, Ravenscroft et al. 2013)	Am J Hum Genet.
Nebulin (NEB) mutations in a childhood onset distal myopathy with rods and cores uncovered by next generation sequencing	(Scoto, Cullup et al. 2013)	Eur J Hum Genet.
Congenital myasthenic syndromes due to mutations in ALG2 and ALG14	(Cossins, Belaya et al. 2013)	Brain
Isoprenoid synthase domain containing gene mutations are a common cause of congenital and limb girdle muscular dystrophies	(Cirak, Foley et al. 2013)	Brain
Pontocerebellar hypoplasia type 1: clinical spectrum and relevance of EXOSC3 mutations	(Rudnik-Schoneborn, Senderek et al. 2013)	Neurology
Mutations in B3GALNT2 cause congenital muscular dystrophy with hypoglycosylation of alpha-dystroglycan	(Stevens, Carss et al. 2013)	Am J Hum Genet.
Mutations in BICD2 cause dominant congenital spinal muscular atrophy and hereditary spastic paraplegia	(Oates, Rossor et al. 2013)	Am J Hum Genet.
Mutations in GDP-mannose pyrophosphorylase B cause congenital and limb-girdle muscular dystrophies associated with hypoglycosylation of alpha-dystroglycan	(Carss, Stevens et al. 2013)	Am J Hum Genet.
Mutations in KLHL40 are a frequent cause of severe autosomal-recessive nemaline myopathy	(Ravenscroft, Miyatake et al. 2013)	Am J Hum Genet.
NDUFA4 mutations underlie dysfunction of a cytochrome C oxidase subunit linked to human neurological disease	(Pitceathly, Rahman et al. 2013)	Cell Rep.
SGK196 is a glycosylation-specific O-mannose kinase required for dystroglycan function	(Yoshida-Moriguchi, Yu et al. 2010)	Science
An RYR1 mutation associated with malignant hyperthermia is also associated with bleeding abnormalities	(Lopez, Byrne et al. 2016)	Sci Signal.
Adenovirus-mediated expression of myogenic differentiation factor 1 (MyoD) in equine and human dermal fibroblasts enables their conversion to caffeine-sensitive myotubes	(Fernandez-Fuente, Martin-Duque et al. 2014)	Neuromuscul Disord.
RyR1 deficiency in Congenital myopathies dDisrupts excitation-contraction coupling	(Zhou, Rokach et al. 2013)	Hum Mutat.
Establishment of a human skeletal muscle-derived cell line: biochemical, cellular and electrophysiological characterization	(Rokach, Ullrich et al. 2013)	Biochem J.

### 1.11 Biomarker development and natural history studies

Collection of biomaterials for biomarker- and natural history studies are two core translational activities of the biobank and current research is dedicated to identification of potential protein biomarkers for prediction of disease course, complications or response to therapy in NMDs. The use of -omics and other technologies have resulted in a considerable number of publications, many of which proposed several circulating biomarkers for the early detection and diagnosis in NMDs. These findings hold promise for the development of new clinical management for these patients. Examples of these studies include MMP-9 [16]; CA3, MYL3, MDH2, ETFA [17]; ADAMTS5 [18]; CA1, FBLN1, GC, GSN [19], miRNAs in Duchenne muscular dystrophy [20] and in spinal muscular atrophy, with cross validation in SMA mouse models following therapeutic intervention [21] (Table 5). In the same way, obtaining maximum value to support drug development programs depends on conducting natural history studies before potential therapeutic agents are identified for development. Well conducted natural history studies are important for understanding the range of phenotype manifestations, the aetiology and progression of rare diseases.

**Table 5:** Publications arisen from biomarker studies.

Title (biomarker identification)	Reference	Journal
Altered Levels of MicroRNA-9, -206, and -132 in Spinal Muscular Atrophy and Their Response to Antisense Oligonucleotide Therapy	(Catapano, Zaharieva et al. 2016)	Mol Ther Nucleic Acids
Global serum glycoform profiling for the investigation of dystroglycanopathies & congenital disorders of glycosylation	(Heywood, Bliss et al. 2016)	Mol Genet Metab Rep.
Altered levels of microRNA-9, -206, and -132 in spinal muscular atrophy and their response to antisense oligonucleotide	(Catapano, Zaharieva et al. 2016)	Therapy. Mol Ther Nucleic Acids
Correlation of utrophin levels with the dystrophin protein complex and muscle fibre regeneration in Duchenne and Becker muscular dystrophy muscle biopsies	(Janghra, Morgan et al. 2016)	PLoS One
Deep RNA profiling identified CLOCK and molecular CLOCK genes as pathophysiological signatures in collagen VI myopathy	(Scotton, Bovolenta et al. 2016)	J Cell Sci.
204th ENMC international workshop on biomarkers in Duchenne muscular dystrophy 24–26 January 2014, Naarden, The Netherlands	(Ferlini, Flanigan et al. 2015)	Neuromuscul Disord.
Epigenetic changes as a common trigger of muscle weakness in congenital myopathies	(Rokach, Sekulic-Jablanovic et al. 2015)	Hum Mol Genet.
The EuroBioBank Network: 10 years of hands-on experience of collaborative, transnational biobanking for rare diseases	(Mora, Angelini et al. 2015)	Eur J Hum Genet.
Dystrophin quantification: biological and translational research implications	(Anthony, Arechavala-Gomeza et al. 2014)	Neurology
Muscle proteomics reveals novel insights into the pathophysiological mechanisms of collagen VI myopathies	(De Palma, Capitanio et al. 2014)	J Proteome Res.
Affinity proteomics within rare diseases: a BIO-NMD study for blood biomarkers of muscular dystrophies	(Ayoglu, Chaouch et al. 2014)	EMBO Mol Med.
Dystromirs as serum biomarkers for monitoring the disease severity in Duchenne muscular Dystrophy	(Zaharieva, Calissano et al. 2013)	PLoS One
Flow cytometry for the analysis of a-dystroglycan glycosylation in fibroblasts from patients with dystroglycanopathies	(Stevens, Torelli et al. 2013)	PLoS One
Dystromirs as serum biomarkers for monitoring the disease severity in Duchenne muscular dystrophy	(Zaharieva, Calissano et al. 2013)	PLoS One

### 1.12 Translational research

The MRC Biobank has been instrumental in providing cells for muscle stem cell research, focused on the characterisation of optimal stem cells, as well as, in development of viral gene therapy vector for autologous cell transplantation in muscular dystrophy and the further characterisation of molecular pathways relevant for muscle stem cell function (Table 6). In addition, cells from the MRC Centre have supported studies for target identification for splicing modification or allele selective silencing using antisense oligonucleotides for collagens VI-related muscular dystrophy, *RYR1*-related myopathies, *SLPTC1* neuropathies and spinal muscular atrophy, as well as, for gene editing using CRISPR/Cas9 (unpublished data). Fibroblasts lines obtained from patients with DMD were used to generate human induced pluripotent stem cells (iPSCs) cardiomyocytes harbouring DMD mutations to enable therapeutic approaches such as gene therapy and exon skipping to be tested in human cardiomyocytes [22]. Other fibroblasts cell lines obtained from patients' biopsies carrying the most common mitochondrial mutations were subjected to non-integrating Sendai virus mediated reprogramming [23]. Established stable iPSCs lines were validated for positive expression of pluripotency factors and formation of three germ layers: mesoderm, endoderm and neuroectoderm. Validated cell lines were subsequently karyotyped to exclude lines with chromosomal rearrangements. Validated human iPSCs has been successfully differentiated into multinucleated skeletal muscles via stepwise differentiation protocol. The differentiation culture system contains defined myogenic factors, which allow recapitulating developmental stages of myogenesis. Each stage of differentiation including: late presomatic mesoderm specification, myoblast formation, cell cycle withdrawal, commitment and cell fusion to form multinucleated myotubes is assessed via quantification of differentiation markers at the RNA and protein level. Maturation is performed by reducing knock-out serum replacement or supplementing media with selected serum components. Current work, in collaboration with UCL Eastman Dental Institute specialising in materials and tissue engineering, focuses on transferring monolayer culture conditions into scaffold-facilitated 3D culture.

**Table 6:** Publications on translational research supported by the biobank.

Title (translational research)	Reference	Journal
Autologous skeletal muscle derived cells expressing a novel functional dystrophin provide a potential therapy for Duchenne muscular dystrophy	(Meng, Counsell et al. 2016)	Sci Rep.
Spell checking nature: versatility of CRISPR/Cas9 for developing treatments for inherited disorders	(Wojtal, Kemaladewi et al. 2016)	Am J Hum Genet.
The effect of the muscle environment on the regenerative capacity of human skeletal muscle stem cells	(Meng, Bencze et al. 2015)	Skelet Muscle
Gene expression profiling identifies molecular pathways associated with collagen VI deficiency and provides novel therapeutic targets	(Paco, Kalko et al. 2015)	PLoS One
The transgenic expression of LARGE exacerbates the muscle phenotype of dystroglycanopathy mice	(Whitmore, Fernandez-Fuente et al. 2014)	Hum Mol Genet.
The human desmin promoter drives robust gene expression for skeletal muscle stem cell-mediated gene therapy	(Jonuschies, Antoniou et al. 2014)	Curr Gene Ther.
BMI1 enhances skeletal muscle regeneration through MT1-mediated oxidative stress protection in a mouse model of dystrophinopathy	(Di Foggia, Zhang et al. 2014)	J Exp Med.
Mutation of the human mitochondrial phenylalanine-tRNA synthetase causes infantile-onset epilepsy and cytochrome c oxidase deficiency	(Almalki, Alston et al. 2014)	Biochim Biophys Acta.
Targeted exon skipping to correct exon duplications in the dystrophin gene	(Greer, Lochmuller et al. 2014)	Mol Ther Nucleic Acids
Antisense suppression of donor splice site mutations in the dystrophin gene transcript	(Fletcher, Meloni et al. 2013)	Mol Genet Genomic Med.
Mitochondrial DNA deletions in muscle satellite cells: implications for therapies	(Spendiff, Reza et al. 2013)	Hum Mol Genet.
Exon skipping and gene transfer restore dystrophin expression in human induced pluripotent stem cells-cardiomyocytes harbouring DMD mutations	(Dick, Kalra et al. 2013)	Mol Ther Nucleic Acids
Two new protocols to enhance the production and isolation of human induced pluripotent stem cell lines	(Dick, Matsa et al. 2011)	Stem Cell Res.

### 1.13 Collaboration with industry

The MRC Biobank has been collaborating with an increasing number of pharmaceutical companies involving biomarker studies. Biobank samples have been provided to industrial partners such as Pfizer [24], [25] and Bristol-Myers-Squibb for identification of serum and urine biomarkers (manuscript submitted). Due to high number of requests, the biobank has improved its access procedures and recently introduced a ‘cost recovery plan’. Cost recovery involves the sharing of costs incurred for sample collection and management. When third parties apply to use the stored samples, they may be asked to provide money to the biobank to help cover the sample collection and management costs. These charges are applied to reduce some of the storage costs and support the availability of funds for ongoing sample collection and preservation; however, no profit is generated from the use or storage of biobank samples. The charges are under regular review to ensure that it is being used appropriately and fairly.

### 1.14 Additional financial and institutional support

Due to limited funding and increased interest in biobank as a repository for multi-centre studies, the biobank is seeking additional support from internal and external sources. For instance, the Jain Foundation has funded technician time and consumables to process and store large numbers of samples from their multi-centre outcome study. Several research groups that use the services of the biobank regularly include funding for biobanking in their grant applications which cover some of the biobank running cost.

## 2 Discussion

Given the rarity, heterogeneity and complexity of neuromuscular disorders, as well as considerable morbidity and mortality, there is great need for establishing dedicated infrastructures to facilitate research. In cooperation with other European centres for neuromuscular diseases and associated biobanks, the MRC Biobank UK may provide an example that such facilities enable and promote research leading to improve diagnosis and treatment. Membership in European networks and coordinated projects such as EuroBioBank, BBMRI and RD-Connect strengthens visibility and supports quality assurance, connecting the biobank and researchers worldwide. MRC Biobank samples and data are a relevant and important source for researchers working in academia and industry, particularly to improve the diagnosis and treatment of patients with rare neuromuscular disorders, therefore contributing to the IRDiRC mandate. MRC Biobank activities have led to the collection of more than 13,000 anonymised, quality-assured samples resulting in high profile research publications and supporting translational research. There are clear indications for an increase in collaboration, global networking and openness using the biobank service. In the past few years the MRC Biobank for Neuromuscular Diseases in the UK has facilitated successful research in the field of neuromuscular disorders. Within the first years of its establishment, the biobank surpassed all the originally established milestones and evolved into an essential source for collection, management and sharing of high quality samples and data. Undoubtedly without the biobank as a central infrastructure with a secure custodianship for data and samples, funds would be spent on unnecessary duplication of collection and management of samples or research projects. Moreover, samples and data may get lost when funding for certain projects expires or the researcher moves on to a different project or research group. In this context, the biobank has not only the duty for the guardianship of biospecimens and data for current studies and projects but also for providing a platform for the benefit of progress in research for future generations. The success of the first nine years of biobank activities confirms that continuous support and sustainability of the biobanks in the field of rare diseases is an indispensable necessity, starting from the collection of these scarce and sometimes very unique samples through operational level and standardisation of biospecimen processing, long-term sample storage, protection and custody of the collected biomaterials and data management. Despite financial support from internal resources of both centres and partial funding acquired from external sources, the long-term sustainability of the biobanks remains a major challenge. Despite the recent progress in the broad-scaled analysis of proteins in body fluids, there is still a lack in protein profiling approaches for biomarkers of rare diseases which emphasises the crucial role of biobank work in continuous sample and data collection of patients with rare diseases. One of the main challenges using biobank samples is possible variability in collection and processing of body fluids including urine and blood which can have dramatic effects on results and analytical reliability. Access to high-quality specimens that are collected and processed in a standardised way reduces potential bias and false results. Therefore, it is essential that standard operating procedures are in place to minimise the degree of discrepancy in the collection and to reduce the variability. However, this is not always possible, in particular, the quality of body fluids can be affected by various parameters that need to be controlled during sample collection, storage and sample preparation such as food intake prior to sample collection, the time between collection and processing, temperature, and even the way the blood samples have been taken. These factors can significantly influence the quality of samples and produce biased findings in their proteomic profiles. The biobank is continuing to improve the quality of samples, in particular for biomarker studies. Biobank sample management and quality may benefit from the implementation of a laboratory accreditation system according to an international standard laboratory quality control system model such as ISO 9001. Moreover, international companies are showing a growing interest in biobank samples and the biobank is continuing to collect and expand a broader range of very rare disease types and samples. Future studies would also benefit from including more cohorts with longitudinal follow-up samples which provides the opportunity to support drug development, clinical trials and gene therapy approaches giving us hope to ultimately find cure for neuromuscular and other rare diseases.

## Appendix Supplementary material

Appendix S1 CONSENT FORM for Adult – London. Appendix S1

Appendix S2 Transfer record – London. Appendix S2

Appendix S3 Consent form – Newcastle. Appendix S3

Appendix S4 Biopsy referral form – Newcastle. Appendix S4

Appendix S5 Blood referral form – Newcastle. Appendix S5

Appendix S6 Biobank sample request – London. Appendix S6

Appendix S7 Biobank sample request (non-relevant material) – London. Appendix S7

Appendix S8 Sample application form – Newcastle. Appendix S8
