# Supplementary material for: MRC Centre Neuromuscular Biobank (Newcastle and London): Supporting and facilitating rare and neuromuscular disease research worldwide
Source: Neuromuscul Disord. 2017 Nov;27(11):1054–64. doi: 10.1016/j.nmd.2017.07.001 (PMC5678293; doi:10.1016/j.nmd.2017.07.001)
Supplement: Appendix S1 — CONSENT FORM for Adult – London. [file mmc1.pdf]

CONSENT FORM for Adults

Title of the Research Project: Setting up of a rare diseases biological samples bank (Biobank) for research to facilitate pharmacological, gene and cell therapy trials in neuromuscular disorders (NMD)

Sponsor Protocol Number: 08ND17
Investigator: Prof Francesco Muntoni

Biobank ID:

Patient details:

Name:
D.O.B:
Hospital #:
Gender:

**1**	I confirm that I have read and understand the information sheet dated 13th July 2015 (version 8) for the above study. I have had the opportunity to consider the information, ask questions and have had these answered satisfactorily.		
**2**	I consent to taking part in the above study.		
**3**	I understand my participation is voluntary and I am free to withdraw at any time, without giving any reason, without my medical care or legal rights being affected.		
**4**	I understand that relevant sections of any of my medical notes and data collected during the study may be looked at by employees from Regulatory Authorities or from the hospital, where it is relevant to my taking part in this research. I give permission for these individuals to have access to my records.		
**5**	I consent that anonymised information on the sample/s and data is provided to scientists through publicly accessible catalogues and databases such as www.eurobiobank.org		
**6**	I agree to my GP being informed of my participation in the study.		
**7**	I agree for a sample of the following tissues to be obtained:	**a)** Skin	
		**b)** Blood	
		**c)** Muscle	
		**d)** Bone cartilage	
		**e)** Nerve	
		**f)** Urine	
		**g)** Cerebrospinal fluid	
**8**	I agree for the tissue obtained to be kept indefinitely in the MRC Neuromuscular Centre Biobank. These samples will constitute a ‘gift’ to the research team, who will not generate any financial gains from this research.		
**9**	I agree to the samples being used within the UK and internationally for future ethically approved research projects into the causes of genetic conditions and treatments for such conditions.		
**10**	I understand that the results from future research may not have any direct implications for myself or my family.		
**11**	I agree to the samples being used by a commercial organisation for the purposes of research		
**12**	I give permission for my donated samples to potentially be used to generate iPS cells. I understand the iPS cells may be generated, stored and distributed for research by other biobanks. I understand that if I withdraw my consent, any iPS cells that have already been created from the donated samples will not be destroyed and any information about them will be retained.		

Please initial boxes 1-12 and sign below to indicate agreement

Signature of patient:

Name: ______________________________

Date: ______________________________

Signature: ___________________________

Signature of person taking consent:

Name: ______________________________

Date: _______________________________

Signature: ___________________________

1 copy for the patient, 1 copy in the hospital notes, original to be kept in the PI's site file

Information for adult patients about “Setting up of a rare diseases biological samples bank (Biobank) for research”

We would like to invite you to take part in a research study. Before you decide whether you wish to participate or not, it is important for you for understand why the research is being done and what it will involve. Please read the following information carefully and discuss it with relatives, friends and your GP, if you wish. If there is anything that is not clear, or if you have any further questions, please do not hesitate to ask us. Take time to decide whether you would like to take part or not.

If you do decide to participate, please let us know beforehand if you have been involved in any other study during the last 6 months. If you decide not to take part, your treatment will not be affected by your decision. You are free to withdraw at any time without explanation and any subsequent treatment will not be affected.

What is the purpose of the study?

Recent advances in the field of DNA research are allowing scientists to design new therapeutic options for many genetic diseases. Current and future research might help us to understand genetic diseases even better and therefore improve the ability to develop treatments for people suffering from rare diseases. For this reason, we would like to create a ‘bank’ of tissue samples, biological fluids, genetic material and cells obtained from blood, skin, muscle, nerve, urine, cerebrospinal fluid and bone cartilage from healthy people as well as from those with diagnosed diseases, for use in future research.

Why have I been chosen?

You have been chosen because you have a rare neuromuscular disorder for which there is currently no cure. Your condition makes your muscles weak and you may have problems with standing or walking.

Do I have to agree to take part?

It is up to you to decide whether you want to take part or not. If you do agree, you will be given this information to keep and be asked to sign a consent form. Even if you do decide to take part and then change your mind, you are still free to withdraw your samples from the laboratory at any time and without giving further explanation as participation in this project is voluntary. This will not affect the standard of care that you receive.

What will happen to me if I agree to take part?

If you agree to participate in this project, we will take the one or several or all of the following samples from you:

- ☐ A sample of blood (approximately 4 teaspoons) **and/or**
- ☐ A urine sample **and/or**
- ☐ A skin sample (the size of the head of matchstick) **and/or**
- ☐ A small piece of muscle (the size of an orange pip) **and/or**
- ☐ A nerve sample (half the size of an orange pip) **and/or**
- ☐ A cerebrospinal fluid sample (up to 2 teaspoons) **and/or**
- ☐ A small piece of bone cartilage (half the size of an orange pip).

If you are coming in for an outpatient hospital appointment, we will only ask for blood, urine and/or skin sample. If you are coming in for an operation or biopsy the blood, skin, muscle, nerve, cerebrospinal fluid and/or bone cartilage will be taken at the site of planned surgery. We will only ask to collect a nerve sample if your doctor has already arranged this procedure for diagnostic reasons. We will only ask to collect cerebrospinal fluid if you are having spinal surgery or a lumbar puncture as recommended by your doctor.

You may wish for only one, or some and not all, samples to be taken and this is possible.

Please note:
No lifestyle restrictions are required to take part in this research. You will be able to eat and drink as normal as would the protocol dictate following the operation and should take all your usual medications.

What will happen to my samples and data if I agree to take part?

We will freeze down some of the samples (such as a small piece of muscle) and also establish cultures of cells and genetic material such as DNA from the tissue(s) and/or blood you have decided to donate to us. We may also prepare plasma and serum from blood.

The samples and data will be anonymised and used in ethically approved research projects to facilitate research into rare neuromuscular diseases within the UK and internationally. The research will be conducted by scientists who work for a recognised academic institution or hospital. The samples may also be used by a commercial organisation but only for the purposes of research if you consent to this. Research may involve genetic analysis of the anonymised samples. All requests for samples and data are reviewed by a Biobank committee to assess the suitability of the research. Legal agreements will be established with researchers to ensure the samples and data are only used for the research specified and within the terms of your consent.

Samples will be stored indefinitely. Should you decide to withdraw your consent or in the unlikely event that the samples are damaged in any way, the unused samples (both stored with the Biobank and distributed to other researchers) will be disposed in a respectful manner and incinerated. No further data will be collected if you have withdrawn your consent but it will not be possible to withdraw research data already obtained from the samples. Please note that any iPS cells already generated and associated data will not be destroyed and may continue to be used in research studies. This is because the process of making iPS cells is time-consuming and expensive. You are free to refuse consent for the generation of iPS cells from your donated samples.

Induced Pluripotent Cells (iPS cells)

An iPS cell is a type of ‘pluripotent’ stem cell, which means that it can grow into most specialised cell types in the human body, such as muscle, nerve or liver cells. iPS cells are generated from samples of tissue such as blood and skin and ‘induced’ in the laboratory to be pluripotent. iPS cells can be cultivated to allow them to survive indefinitely. Human iPS cells are useful research tools, especially for studying the effect of genes on disease risks and for development of new drugs and methods. The iPS cells generated from the samples you donate will only be used for research. They will never be used to clone a new human being, and will never be transplanted into a human being.

The Biobank is working collaboratively with stem cell banks such as the European Bank for Induced Pluripotent Stem Cells (www.ebisc.org) and StemBANCC (www.stembancc.org). The Biobank may prepare cells from your donated samples and these cells may then be sent to stem cell banks to generate and store iPS cells. These cells will be anonymised and stem cell banks will not have access to your personal information. The stem cell banks may then distribute the iPS cells to researchers, where this is compliant with all relevant legal rules and regulations and approved as necessary by a research ethics committee.

You must explicitly give consent for generation of iPS cells from your samples by initialling the last box on the consent form. If you would prefer your samples were not used to generate iPS cells, please do not initial the last box.

What about confidentiality?

When you are given information about your taking part in research, you should be told that people other than your doctors would need access to your medical records. Any of the researchers who see your records have to follow the same confidentiality guidelines as other hospital staff. Information about you will not leave the Biobank unless it has been given an anonymised code and personally identifiable information such as your name, date of birth and hospital number have been removed. This anonymised data may be made available on publically accessible sample catalogues and databases such as eurobiobank.org to facilitate scientific research. Unless you have provided specific authorization your personal results will not be made available to third parties such as employers, governmental organizations, insurance companies or educational institutions. This also applies to other members of your family. However, if you do decide to take part in this research, your family doctor (GP) will be advised of your participation and of your treatment, unless you have any objections to this.

What are the possible risks of taking part in the study?

There is no additional risk to you in participating in this project. Any risks are associated with the surgery itself and your doctor will discuss these with you.

No extra tissue will be taken during the operation or biopsy other than what would normally be collected for diagnostic reasons or discarded during surgery.

What are the possible benefits of taking part?

There will not necessarily be an immediate clinical benefit to you as a result of your participation in this research. However, we hope that the information obtained from this study will enable us to improve the knowledge and help us to treat you and other individuals with rare neuromuscular disorders better in the future. Information gathered may be useful for you or your relatives but likely not in the immediate future. All the results regarding the work performed as part of this study, from scientific publications to intellectual properties derived from new discoveries, will be handled by the University sponsoring the study. As such you will not be entitled to a share of any possible profits resulting from data/products derived from the use of the tissue donated.

What if something goes wrong?

There are no special compensation arrangements if you are harmed by taking part in this research project. If you are harmed due to someone’s negligence, then you may have grounds for legal action, but you may have to pay for it. Regardless of this, if you wish to complain or have concerns about any aspect of your treatment, you should contact the Trust’s Complaints Co-ordinators.

What will happen to the results of the research study?

The results from the research study may be used to write an article to be submitted for publication in scientific or medical journals. It is also likely that the results of this study will be presented at meetings. However, please be reassured that you will not be identified in any such reports or publications as data obtained will only be published in an anonymous and aggregated way, that is to say, as percentages or numerical data without identification of the participant, never in an individualised way to prevent your identification.

Who is organising and funding the research?

The Department of Health, the Medical Research Council and different charities (Muscular Dystrophy Campaign; Welcome Trust) are funding the research. The investigators will not receive any financial reward for recruiting patients for this study.

Who monitors the researchers?

Whether a research study is a large drug trial sponsored by a pharmaceutical company, or a small study being done by a student, there are certain checks in place. Before any research begins, it will have been approved by a Research Ethics Committee. The application to the Research Ethics Committee will describe everything that will take place in the study and how it will be done. There is an independent Research Advisory Board that periodically reviews the progress of the research project.

The London – West London & GTAC Research Ethics Committee (formerly known as the Hammersmith, Queen Charlotte's and Chelsea Research Ethics Committee) has reviewed and approved this study.

For further information, please contact:

If you have any questions or concerns, please do not hesitate to contact Professor Francesco Muntoni at the following address:

Professor Francesco Muntoni
Dubowitz Neuromuscular Unit
UCL Institute of Child Health
30 Guilford St
London, WC1N 1EH

If you agree to participate in this research, please sign the attached consent forms. You will be given a copy of the information sheet and a signed consent form to keep for your records.
