# Supplementary material for: MRC Centre Neuromuscular Biobank (Newcastle and London): Supporting and facilitating rare and neuromuscular disease research worldwide
Source: Neuromuscul Disord. 2017 Nov;27(11):1054–64. doi: 10.1016/j.nmd.2017.07.001 (PMC5678293; doi:10.1016/j.nmd.2017.07.001)
Supplement: Appendix S2 — Transfer record – London. [file mmc2.pdf]

FORM F3: TRANSFER RECORD: SAMPLE FROM DIAGNOSTIC ARCHIVE TO BIOBANK

SAMPLE INFORMATION			
**Request date**			
**Sample type**		**Sample ID**	
**Consent**	**Required?**<br>☒ Yes →<br>☐ No →	**Consent type**<br>☐ GOSH/Hammersmith ☐ Referral ☐ Biobank<br>☐ Existing holding, date received / /<br>☐ No consent list checked	
**Diagnosis**			
**Date discussed at MDT**		**Date of pathology report**	
**Quantity of tissue available**			
**Project requesting tissue**			

APPROVAL DECISION	
**Tissue release**	___ Approved<br>___ Not approved
**Tissue released to**	___ Biobank – any ethically approved project<br>___ Condition – specified project
**Quantity of tissue to transfer**	___ All available<br>___ Portion (specify)…………………………………………………
**Date approved**	
**Signature**	Dr Rahul Phadke
**Any comments**	
