# Supplementary material for: MRC Centre Neuromuscular Biobank (Newcastle and London): Supporting and facilitating rare and neuromuscular disease research worldwide
Source: Neuromuscul Disord. 2017 Nov;27(11):1054–64. doi: 10.1016/j.nmd.2017.07.001 (PMC5678293; doi:10.1016/j.nmd.2017.07.001)
Supplement: Appendix S3 — Consent form – Newcastle. [file mmc3.pdf]

Newcastle MRC Centre Biobank for Rare and Neuromuscular Diseases

Adult Consent Form

V3 14th March 2016

Title of Research Project: Newcastle MRC Centre Biobank for Rare and Neuromuscular Diseases

Principal Investigator: Professor Hanns Lochmüller

Patient Details:

Name:
DOB:
Hospital No:

Please initial box

1.	I confirm that I have read and understand the information sheet dated…..version….. for the above study. I have had the opportunity to consider the information it contains. The risks and benefits of my participation have been discussed with me. I have had the chance to ask questions. These questions have been answered to my satisfaction.		
2.	I understand that my taking part is voluntary. I am free to withdraw at any time, without giving any reason and without affecting my present or future medical treatment.		
3.	I understand that relevant sections of any of my medical notes and data collected during the study may be looked at by individuals from Regulatory Authorities or from the NHS Trust, where it is relevant to my taking part in this research. I give permission for these individuals to have access to my medical records.		
4.	I give permission for my medical information to be stored.		
5.	I agree that anonymised information on the sample/s is provided to scientists through publicly accessible databases such as www.eurobiobank.org and www.rd-connect.eu		
6.	I agree for a sample of the following tissues to be obtained:	a. Blood	
		b. Urine	
		c. Saliva	
		d. Skin	
		e. Muscle	
		f. Nerve	
		g. Other: specify__________________	
7.	I agree for tissue and data obtained to be kept indefinitely in the Newcastle MRC Centre Biobank for Rare and Neuromuscular Diseases. These samples will constitute a “gift” to the research team, who will not generate any financial gains		

	from this research.	
8.	I agree to the samples being used for approved researcher projects into the causes and treatments of genetic and neuromuscular conditions now, in the future and after my death. I understand that this research may include sequencing of my genome.	
9.	I give permission for my sample to be used in experiments using rodents (rats and mice)	
10.	I give permission for my samples and anonymised clinical information to be sent to centres outside of the UK for research purposes	
11.	I give permission for my samples to be used by commercial partners (e.g. pharmaceutical companies) for research purposes.	
12.	I understand that the results from future research may not have any direct implications for myself or my family.	
13.	I understand that I will not be told the results of any test which may be carried out on my samples unless relevant for my medical condition.	
14.	I consent to take part in this research project.	

Patient	Date	Signature
Person taking consent	Date	Signature

**Number of Samples Taken**		
**Purpose of Sample (please Circle)**	**BIOBANK**	**DIAGNOSTIC**
