# Supplementary material for: MRC Centre Neuromuscular Biobank (Newcastle and London): Supporting and facilitating rare and neuromuscular disease research worldwide
Source: Neuromuscul Disord. 2017 Nov;27(11):1054–64. doi: 10.1016/j.nmd.2017.07.001 (PMC5678293; doi:10.1016/j.nmd.2017.07.001)
Supplement: Appendix S4 — Biopsy referral form – Newcastle. [file mmc4.pdf]

NCL-ID:

Newcastle MRC Centre Biobank for Rare and Neuromuscular Diseases

PI: Prof. Hanns Lochmüller
Institute of Genetic Medicine
Newcastle University
Central Parkway
Newcastle upon Tyne
NE1 3BZ
+44 191 241 8602
hanns.lochmuller@ncl.ac.uk

Biopsy Referral Form

Forename | **Male** ☐ | **Female** ☐

Surname | **GC Nº**

DOB

Complete if sample is part of a study

Study Title

Patient ID | **Study Visit**

Muscle ☐ | **Skin** ☐ | **Nerve** ☐ | **Other**

Diagnosis / Mutation

Relevant Clinical Details

Ambulant ☐

Ambulant Long Distances (Wheelchair Use) ☐

Ambulant Short Distances (Wheelchair Use) ☐

Non-Ambulant (Wheelchair Bound) ☐

Consultant | **Biopsy Performed by**

Consent Obtained by | **Date**

Biobank Use Only

Date Proliferated: | Additional Information:

Date Frozen:

Storage Location:
