# Supplementary material for: MRC Centre Neuromuscular Biobank (Newcastle and London): Supporting and facilitating rare and neuromuscular disease research worldwide
Source: Neuromuscul Disord. 2017 Nov;27(11):1054–64. doi: 10.1016/j.nmd.2017.07.001 (PMC5678293; doi:10.1016/j.nmd.2017.07.001)
Supplement: Appendix S6 — Biobank sample request – London. [file mmc6.docx]

Date of request Request #

Ethics# MTA/IDT#

Approved? YES / NO

Date approved

Date supplied Initial

Courier

Airwaybill #

Date receipted Initial

- **Complete one form per project**
- **Append a copy (in English) of your project’s**

**ethical approval and protocol**

- **Return to:**

Vaishnavi Manoharan

Rm 127, Dubowitz Neuromuscular Unit

UCL Institute of Child Health

30 Guilford St, London WC1N 1EH

Ph. 0207 905 2221

[v.manoharan@ucl.ac.uk](mailto:v.manoharan@ucl.ac.uk)

| **YOUR DETAILS** | | |
| --- | --- | --- |
| **Primary contact** | | **Principal Investigator** |
| **Name** |  |  |
| **Address** |  |  |
| **Phone** |  |  |
| **Email** |  |  |

- **Please note:** By completing and submitting this form you agree to abide by the Biobank Access Policy

| **PROJECT DETAILS** | |
| --- | --- |
| **Title of study** |  |
| **Ethics approval number** (append copy) |  |
| **Ethics expiry date** (dd/mm/yyyy) |  |
| **Scientific Background** |  |
| **Aims of study** |  |
| **Investigation plan** |  |
| **Who is the research funded by?**  **Is the grant peer-reviewed?** |  |
| **Likelihood of the research producing patentable results?** | **Expected  Not expected** |

| **SAMPLES REQUESTED**  **If you know the sample IDs, please fill in the table on page 3** | |
| --- | --- |
| **Diagnosis** |  |
| **Type of sample and total number of samples required** |  |
| **Minimum quantity of each sample required** |  |

| **Sample type** | **Sample ID** | **Minimum amount required** | **BIOBANK USE ONLY** | | | | | |
| --- | --- | --- | --- | --- | --- | --- | --- | --- |
|  |  |  | **Consent** | **Diagnosis** | **Sample available?** | **Supply?** | **Amount supplied** | **Date supplied and initials** |
|  |  |  |  |  |  |  |  |  |
|  |  |  |  |  |  |  |  |  |
|  |  |  |  |  |  |  |  |  |
|  |  |  |  |  |  |  |  |  |
|  |  |  |  |  |  |  |  |  |
|  |  |  |  |  |  |  |  |  |
|  |  |  |  |  |  |  |  |  |
|  |  |  |  |  |  |  |  |  |


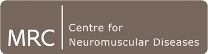


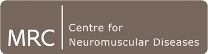


| **BIOBANK REVIEW COMMITTEE** | | | |
| --- | --- | --- | --- |
| **Submission date** |  | | |
|  | **Dr Jenny Morgan** | **Dr Rahul Phadke** | **Dr Manju Kurian** |
| **Delegate**  (if committee member unavailable) |  |  |  |
| **Date** |  |  |  |
| **Comments** |  |  |  |
| **Vote** | Approved  Not approved | Approved  Not approved | Approved  Not approved |

| **APPROVAL DECISION** | |
| --- | --- |
| **Majority vote** | Approved  Not approved |
| **Date approved** |  |
| **Notes** |  |
