# Supplementary material for: MRC Centre Neuromuscular Biobank (Newcastle and London): Supporting and facilitating rare and neuromuscular disease research worldwide
Source: Neuromuscul Disord. 2017 Nov;27(11):1054–64. doi: 10.1016/j.nmd.2017.07.001 (PMC5678293; doi:10.1016/j.nmd.2017.07.001)
Supplement: Appendix S7 — Biobank sample request (non-relevant material) – London. [file mmc7.docx]

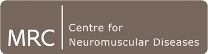


**Date received**

**Request #**

**Date supplied**

**Initial**

- **Return to:**

Vaishnavi Manoharan

v.manoharan@ucl.ac.uk

**Samples are supplied by the biobank on the following conditions:**

- Samples will not be distributed without prior approval by the biobank;
- The MRC CNMD Biobank London will be acknowledged in any publications resulting from the use of samples supplied;
- The biobank will be informed of publications resulting from the use of samples supplied.

| **YOUR DETAILS** | | |
| --- | --- | --- |
| **Primary contact** | | **Principal Investigator** |
| **Name** |  |  |
| **Address** |  |  |
| **Phone** |  |  |
| **Email** |  |  |

| **PROJECT DETAILS** | |
| --- | --- |
| **Title of study** |  |
| **Intended use of sample** |  |

| **Sample type** | **Sample ID** | **BIOBANK USE ONLY** | | | |
| --- | --- | --- | --- | --- | --- |
|  |  | **Consent** | **Date supplied** | **Location** | **Passage** |
|  |  |  |  |  |  |
|  |  |  |  |  |  |
|  |  |  |  |  |  |
|  |  |  |  |  |  |
|  |  |  |  |  |  |
|  |  |  |  |  |  |
|  |  |  |  |  |  |
|  |  |  |  |  |  |
|  |  |  |  |  |  |
|  |  |  |  |  |  |
|  |  |  |  |  |  |
|  |  |  |  |  |  |
|  |  |  |  |  |  |
|  |  |  |  |  |  |
|  |  |  |  |  |  |
