# Supplementary material for: MRC Centre Neuromuscular Biobank (Newcastle and London): Supporting and facilitating rare and neuromuscular disease research worldwide
Source: Neuromuscul Disord. 2017 Nov;27(11):1054–64. doi: 10.1016/j.nmd.2017.07.001 (PMC5678293; doi:10.1016/j.nmd.2017.07.001)
Supplement: Appendix S8 — Sample application form – Newcastle. [file mmc8.docx]

**NEWCASTLE MRC CENTRE FOR NEUROMUSCULAR DISEASES BIOBANK**

**SAMPLE APPLICATION FORM**

RETURN TO: [mojgan.reza@ncl.ac.uk](mailto:mojgan.reza@ncl.ac.uk)

**PLEASE NOTE THAT REQUESTED SAMPLES CAN ONLY BE USED FOR THE PURPOSE STATED ON APPLICATION**

DO NOT INCLUDE PATIENT NAMES ON APPLICATION FORM

**APPLICANT DETAILS**

|  | **RESEARCHER** | **PRINCIPAL INVESTIGATOR** |
| --- | --- | --- |
| **NAME** |  |  |
| **ADDRESS** |  |  |
| **TELEPHONE** |  |  |
| **EMAIL** |  |  |

**PROJECT DETAILS**

| **STUDY TITLE** |  |
| --- | --- |
| **PROJECT TITLE** |  |
| **START DATE** |  |
| **END DATE** |  |
| **LOCATION OF SAMPLE ANALYSIS** |  |
| **ETHICS APPROVAL NUMBER** |  |
| *If Ethics Approval Number IS NOT declared please provide a reason* |  |
| **FUNDING BODY** |  |
| **PROJECT RATIONALE** |  |

**SAMPLE REQUEST**

| **DATE OF REQUEST** |  |
| --- | --- |

| **NUMBER OF SAMPLES REQUIRED** | | | | | | | |
| --- | --- | --- | --- | --- | --- | --- | --- |
| **MYOBLAST** |  | **FIBROBLAST** |  | **PLASMA** |  | **SERUM** |  |
| **URINE** |  | **DNA** |  | **RNA** |  | **OTHER** |  |

If any information about the samples is known please provide below:

| **DIAGNOSIS** |  |
| --- | --- |

If any specific samples are required please provide below:

| **BIOBANK ID (If Known)** | **EXTERNAL ID (If Known)** | **SAMPLE INFORMATION (If Known)** |
| --- | --- | --- |
|  |  |  |
|  |  |  |
|  |  |  |
|  |  |  |
|  |  |  |
|  |  |  |
|  |  |  |
|  |  |  |
|  |  |  |
|  |  |  |
|  |  |  |
|  |  |  |
|  |  |  |

**SHIPMENT DETAILS**

| **COURIER ACCOUNT (TO BE CHARGED)** |  |
| --- | --- |
| **COURIER** |  |
| **SHIPPING ADDRESS** |  |
| **CONTACT TELEPHONE** |  |

**INTERNAL BIOBANK USE ONLY**

| **DATE** | **BIOBANK ID** | **SAMPLE TYPE** | **DIAGNOSIS** | **No VIALS/CONCENTRATION** |
| --- | --- | --- | --- | --- |
|  |  |  |  |  |
|  |  |  |  |  |
|  |  |  |  |  |
|  |  |  |  |  |
|  |  |  |  |  |
|  |  |  |  |  |
|  |  |  |  |  |
|  |  |  |  |  |
|  |  |  |  |  |
|  |  |  |  |  |
|  |  |  |  |  |
|  |  |  |  |  |
|  |  |  |  |  |
|  |  |  |  |  |
|  |  |  |  |  |
|  |  |  |  |  |
|  |  |  |  |  |
|  |  |  |  |  |
|  |  |  |  |  |
|  |  |  |  |  |
|  |  |  |  |  |
|  |  |  |  |  |
|  |  |  |  |  |
|  |  |  |  |  |
|  |  |  |  |  |
|  |  |  |  |  |
|  |  |  |  |  |
|  |  |  |  |  |
|  |  |  |  |  |
|  |  |  |  |  |
|  |  |  |  |  |
|  |  |  |  |  |
|  |  |  |  |  |
|  |  |  |  |  |
|  |  |  |  |  |
|  |  |  |  |  |
|  |  |  |  |  |
|  |  |  |  |  |
|  |  |  |  |  |
|  |  |  |  |  |
